# Association of Inflammatory Cytokine Levels with Extra Glandular Manifestations, Fatigue, and Disease Activity in Primary Sjögren’s Syndrome in Saudi Patients: A Cross-Sectional Study

**DOI:** 10.3390/diagnostics13193036

**Published:** 2023-09-24

**Authors:** Bashaer Alqahtani, Maha Daghestani, Mohammed A. Omair, Esam H. Alhamad, Yusra Tashkandy, Nashwa Othman, Khalid Al Shahrani, Muthurajan P. Paramasivam, Fahidah Alenzi, Rabih Halwani, Fadwa M. Alkhulaifi, Suliman Yousef Alomar

**Affiliations:** 1 Department of Zoology, College of Science, King Saud University, Riyadh 11451, Saudi Arabia; bsmsq@hotmail.com (B.A.); mdaghestani@ksu.edu.sa (M.D.); 2 Rheumatology Unit, Department of Medicine, King Saud University, Riyadh 11451, Saudi Arabia; momair@ksu.edu.sa; 3 Department of Medicine, Division of Pulmonary Medicine, College of Medicine, King Saud University, Riyadh 11451, Saudi Arabia; esamalhamad@yahoo.com; 4 Department of Statistics and Operations Research, College of Sciences, King Saud University, Riyadh 11451, Saudi Arabia; ytashkandi@ksu.edu.sa; 5 Central Laboratory, King Saud University, Riyadh 11451, Saudi Arabia; nashwao@ksu.edu.sa; 6 Rheumatology Division, Department of Medicine, Ad Diriyah Hospital, Ministry of Health, Riyadh 13717, Saudi Arabia; shahrani.md@hotmail.com; 7 Pulmonary Division, Department of Medicine, College of Medicine, King Saud University, Riyadh 11451, Saudi Arabia; drmuthurajan@hotmail.com; 8 Department of Clinical Science, College of Medicine, Princess Nourah bint Abdulrahman University, Riyadh 11671, Saudi Arabia; fmalenzi@pnu.edu.sa; 9 Department of Clinical Sciences, Sharjah Institute for Medical Research (SIMR), College of Medicine, University of Sharjah, Sharjah 27272, United Arab Emirates; rhalwani@sharjah.ac.ae; 10 Biology Department, College of Science, Imam Abdulrahman bin Faisal University, Dammam 34212, Saudi Arabia; falkhulaifi@iau.edu.sa

**Keywords:** primary Sjögren’s syndrome, ESSDAI, ESSPRI, FSS, PGA, PhGA

## Abstract

Background: Primary Sjögren’s syndrome (pSS) is an autoimmune disease that can cause fatigue and extraglandular manifestations (EGMs). pSS is associated with cytokine network dysregulation, which may be related to the immune-mediated destruction of exocrine glands. Objective: We determined cytokine levels and their relationship to EGMs, the European League Against Rheumatism (EULAR) Sjögren’s syndrome disease activity index (ESSDAI), and fatigue in Saudi patients with pSS. Methods: This study was a cross-sectional, single-center study. We included forty-one patients and 71 controls. Serum samples were collected from random healthy people and pSS patients who were followed in the rheumatology and pulmonary clinics of King Saud University Medical City in Riyadh, Saudi Arabia. Levels of the frequently studied cytokines were measured using Luminex xMAP technology. Each ESSDAI score and EGM were recorded, and the Arabic version of the fatigue severity scale (FSS) was applied to assess fatigue. The main outcome measures were cytokine levels in pSS Saudi patients using/not using immune-suppressive medications (ISMs). Results: Thirty-six (87.8%) patients had one or more EGMs, and the mean ESSDAI score was 9.95 ± 7.73. There was a significant decrease in TNFα and IL-21 levels in the pSS group compared to those in the control group (*p* = 0.034 and *p* < 0.001, respectively), whereas IL-12 levels were significantly elevated in the pSS group (*p* = 0.002). Cytokine levels in patients who used ISMs were the same as those in patients who did not use medications. Decreased IL-1β (*p* = 0.014), IL-2 (*p* = 0.035), IL-6 (*p* = 0.014), and IL-35 (*p* = 0.010) levels were observed in patients who had EGMs. Patients who had low disease activity exhibited low IL-10 (*p* = 0.018) and high IFN-α (*p* = 0.049), IFN-β (*p* = 0.049), IL-1β (*p* = 0.006), and IL-35 (*p* = 0.032) levels compared to patients with high disease activity. A negative association between a positive fatigue score and IL-1β (*p* = 0.010), IL-2 (*p* = 0.037), IFN-α (*p* = 0.025), TNFα (*p* = 0.030), IL-17 (*p* = 0.029), IL-12 (*p* = 0.046), and IL-21 (*p* = 0.005) levels was found. Conclusions: Cytokine profiles correlate with EGMs, ESSDAI, and fatigue. Patients with controlled disease activity have a normal cytokine profile that is similar to that of controls.

## 1. Background

Sjögren’s syndrome is an autoimmune disease characterized by exocrine gland destruction due to infiltrated autoreactive lymphocytes and resulting in sicca symptoms [1].

The disease can be classified as primary Sjögren’s syndrome (pSS), occurring without any association with other inflammatory diseases, or secondary Sjögren’s syndrome, in association with another autoimmune disease (e.g., rheumatoid arthritis or systemic lupus erythematosus) [2].

Sjögren’s syndrome affects 1 in 2000 adults and is the second most common systematic rheumatic disease globally after rheumatoid arthritis [3]. Sjögren’s syndrome can cause fatigue, which is a subjective experience that affects many patients with rheumatological conditions (e.g., rheumatoid arthritis; systemic lupus erythematosus) and neurological disorders (e.g., multiple sclerosis; Parkinson’s disease) [4]. Furthermore, pSS can cause extraglandular manifestations (EGMs), including pulmonary, renal, cutaneous, articular, neurological, and hematological involvement [5,6].

The main hypothesis for pSS etiology is based on the fact that it is a multifactorial autoimmune disease, in which several genes influence its development concurrently, with immunological and environmental factors [7]. Environmental factors such as viral infections can trigger heightened activity of the interferon system, which contributes to the development of Sjögren’s syndrome [8].

It is known that pSS is associated with cytokine network dysregulation, which may be related to the immune-mediated destruction of exocrine glands [9]. For example, interferon gamma (IFN-γ) and interleukin-12 (IL-12) induce the expression of the signal transducer and activator of transcription (STAT-4) to help naïve CD4+ T cells differentiate into Th1 lymphocytes, which produce proinflammatory cytokines [10]. Moreover, proinflammatory cytokines, such as interferon alpha (IFN-α), tumor necrosis factor alpha (TNFα), and IL-12, with other cytokines, are overexpressed [11]. Th2 cells, which differentiate after being induced by interleukin-2 (IL-2) and interleukin-4 (IL-4), produce anti-inflammatory cytokines such as interleukin-10 (IL-10) [12].

To date, there are no studies of pSS in the Middle Eastern population or those with Arab ancestries that focus on cytokine levels or their relationship to EGMs. In fact, the association between fatigue and cytokine levels has been poorly investigated [13,14,15] and has not been determined in Arabian ethnicities.

The EULAR Sjögren’s syndrome disease activity index (ESSDAI) measures disease activity in patients with pSS [16], but there are limited data regarding the relationship between fatigue and ESSDAI, and it has not been applied to patients with Arab ancestry [17,18]. Furthermore, there is a lack of research on the relationship between fatigue and ESSDAI, again with no investigation involving those with Arab ancestry.

We hypothesized that patients with pSS with controlled disease activity based on ESSDAI may have a normal cytokine profile similar to that of controls. This study aimed to evaluate proinflammatory and anti-inflammatory cytokine levels and their relationship to EGMs, ESSDAI, and fatigue in Saudi patients with pSS.

## 2. Materials and Methods

### 2.1. Patient Population

This study is part of a larger clinical study of patients with pSS in Saudi Arabia [19,20]. This study had a cross-sectional design and was performed between October 2018 and May 2019, with 117 Saudi male and female volunteers in total who were aged 18–77 years. The subjects were divided into two groups: Group 1, 71 healthy Saudi subjects with pSS autoantibody negativity and no symptoms, representing the cross-section of Saudi society; Group 2, 46 Saudi patients with pSS who were recruited while they were followed in the rheumatology and pulmonary clinics of King Saud University Medical City in Riyadh, Saudi Arabia. The patients fulfilled the American College of Rheumatology/European League Against Rheumatism (ACR/EULAR) classification criteria [21]. The exclusion criteria were a confirmed diagnosis of malignancy, major psychiatric disorder, or presence of end organ failure.

Clinical characteristics, including age, sex, and EGMs, patient global assessment (PGA), physician global assessment (PhGA), ESSDAI, and clinical components of the EULAR Sjögren’s Syndrome Patient Reported Index (ESSPRI), were obtained during clinic visits. ESSDAI = 0 was set as remission, and level < 5 was set as low disease activity [16]. ESSPRI components were calculated individually and as a single factor composed of the mean of three components: pain, fatigue, and dryness. ESSPRI < 5 was considered an acceptable symptom state [16]. The pulmonary hypertension diagnosis was based on an elevated mean pulmonary artery pressure of ≥25 mmHg as determined via resting supine right heart catheterization according to European Society of Cardiology and the European Respiratory Society [22]. No treatment adjustment was made for the patients who took immunsuppressant medications three months prior to blood sampling for cytokine analysis.

### 2.2. Sample Preparation

This study received ethical approval from the Research Ethics Committee of Medical City at King Saud University, and family history was collected. Written informed consent was obtained from all subjects. In addition, 5 mL of blood was collected from patients and controls in a tube containing EDTA. Plasma was separated via centrifugation and aliquoted and stored at −80 °C until further use.

### 2.3. Multiplex Cytokine Assay

Serum levels of the most frequently studied cytokines, including interferon beta (IFN-β), interleukin-1 beta (IL-1β), IL-2, IL-6, IL-10, IL-17, IL-21, IL-35, B-cell activating factor (BAFF), IFN-α, IL-12p40, and TNFα, were measured using bead-based immunofluorescence assay kits (Merck KGaA, Darmstadt, Germany). The kit IDs were HSTCMAG-28SK-04.Hu. HighSe, HCYTOMAG-60K-04. Hum, and HCYP4MAG-64K-02. Hum.

Serum samples were evaluated in duplicate, and the assay was performed in accordance with the kit manufacturer’s instructions. The Luminex^®^ 200™ system (Luminex® 200™, APX10031, ThermoFisher Scientific, Madison, WI, USA), which is a flow cytometry-based instrument, was used for analysis. Each individual microsphere was identified, and the result of its bioassay was quantified based on fluorescent reporter signals.

### 2.4. Fatigue Score Scale (FSS)

The Arabic version of the FSS is a unidimensional questionnaire used to assess fatigue and consists of nine statements that rate the severity of the patient’s fatigue symptoms in the past week in terms of how these symptoms affect the patient’s physical function and motivation. The patients must score each statement from 1 to 7, where 1 indicates strong disagreement and 7 indicates strong agreement. The scale can be calculated via the mean score of all 9 items; a score of <4 indicates that a patient has no fatigue; a score of >4 indicates fatigue, which is more severe with a higher score [23]. The results of this study are reported in accordance with STROBE guidelines [24].

### 2.5. Statistical Analysis

The descriptive characteristics of group variables are expressed as the mean ± standard deviation and presented in the form of individual value plots and box plots. Cytokine levels between the patients and controls were compared using the independent *t*-test. Comparisons between patients using/not using immunosuppressive medications (ISMs) were performed with the Chi-square test.

Subgroup analysis was performed based on disease activity, the presence of EGMs, and the fatigue score. Associations between cytokine levels and fatigue, ESSDAI, and ESSPRI were determined using the Chi-square test. The Spearman rank correlation coefficient was used to assess associations between cytokines and EGMs. Significant differences are indicated by *p*-values of < 0.05. All statistical analyses were performed using Minitab statistical software (version 21.4.0).

## 3. Results

### 3.1. Demographics

Briefly, 46 patients were recruited between October 2018 and May 2019; of these, 41 fulfilled the study criteria and were included in the final analysis of the study. Twenty-three (78%) were women, with a mean (±SD) age of 58.76 ± 12.7 years and disease duration of 4.6 ± 2.28 years (Table 1). Twenty-two (53.6%) patients in this study were treated with the following ISMs: 19 (46.3%), mycophenolate mofetil; 18 (43.9%), prednisolone; 9 (22%), rituximab; 8 (19.5%), macitentan and sildenafil; 3 (7.30%), cyclophosphamide; 2 (4.90%), azathioprine; and 1 (2.4%), hydroxychloroquine and methotrexate. The mean ESSDAI and ESSPRI scores were 9.95 ± 7.73 and 5.17 ± 2.4, respectively, with individual component scores of ESSPRI for dryness (5.23 ± 2.62), fatigue (5.4 ± 2.88), and pain (4.88 ± 3.31). Based on the FSS, the mean score for the patients was 3.75 ± 1.73; 18 (43.9%) patients had a positive test, with a mean score of 5.43 ± 0.76.

### 3.2. EGM

Thirty-six (87.8%) patients had one or more EGM. The most common EGMs were pulmonary and articular, with each affecting 27 patients (65.9%), followed by pulmonary hypertension (19.5%), hematological manifestation (12.2%), cutaneous manifestation (9.8%), peripheral nervous system manifestation (7.3%), renal dysfunction (4.9%), thrombotic events (4.9%), and central nervous system manifestation (2.4%). At the most recent follow-up, none of the patients had cryoglobulinemic vasculitis or lymphoma or developed any other type of malignancy.

### 3.3. Cytokine Profile

**Figure 1:**
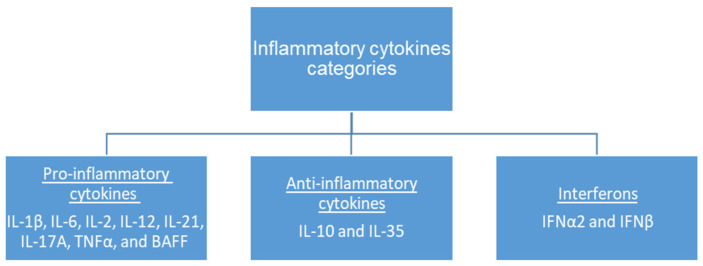
Flow chart representation of inflammatory cytokine categories evaluated in our study. IL-1β: Interleukin-1 beta, IL-6: Interleukin-6, IL-2: Interleukin-2, IL-12: Interleukin-12, IL-21: Interleukin-21, IL-17A: Interleukin-17A, TNFα: Tumor necrosis factor alpha, BAFF: B-cell activating factor, IL-10: Interleukin-10, IL-35: Interleukin-35, IFN-α2: Interferon alpha 2, and IFN-β: Interferon beta.

We divided the cytokines examined in our study into three categories: proinflammatory cytokines (IL-1β, IL-6, IL-2, IL-12, IL-21, IL-17, TNFα, and BAFF), anti-inflammatory cytokines (IL-10 and IL-35), and interferons (IFN-α and IFN-β) (Figure 1).

**Figure 2:**
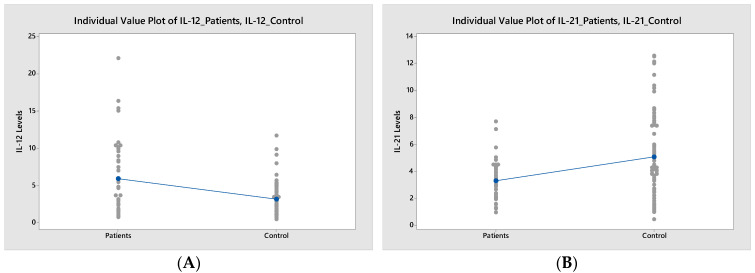
Comparison of cytokine levels in patients with pSS and controls. (**A**). IL-12 (*p* = 0.002). (**B**). IL-21 (*p* < 0.001). C. TNFα (*p* = 0.034). Grey dots represent cytokine values, while blue points and blue lines indicate mean values.

There was a significant decrease in TNFα and IL-21 levels in patients with pSS compared to those in the control group (*p* = 0.034 and *p* < 0.001, respectively) (Table 2). Moreover, the IL-12 levels were significantly elevated in patients with pSS compared to those in the control group (*p* = 0.002) (Figure 2).

Cytokine levels in patients using ISMs were the same as those in patients who were not taking any medication (Table 3).

**Figure 3:**
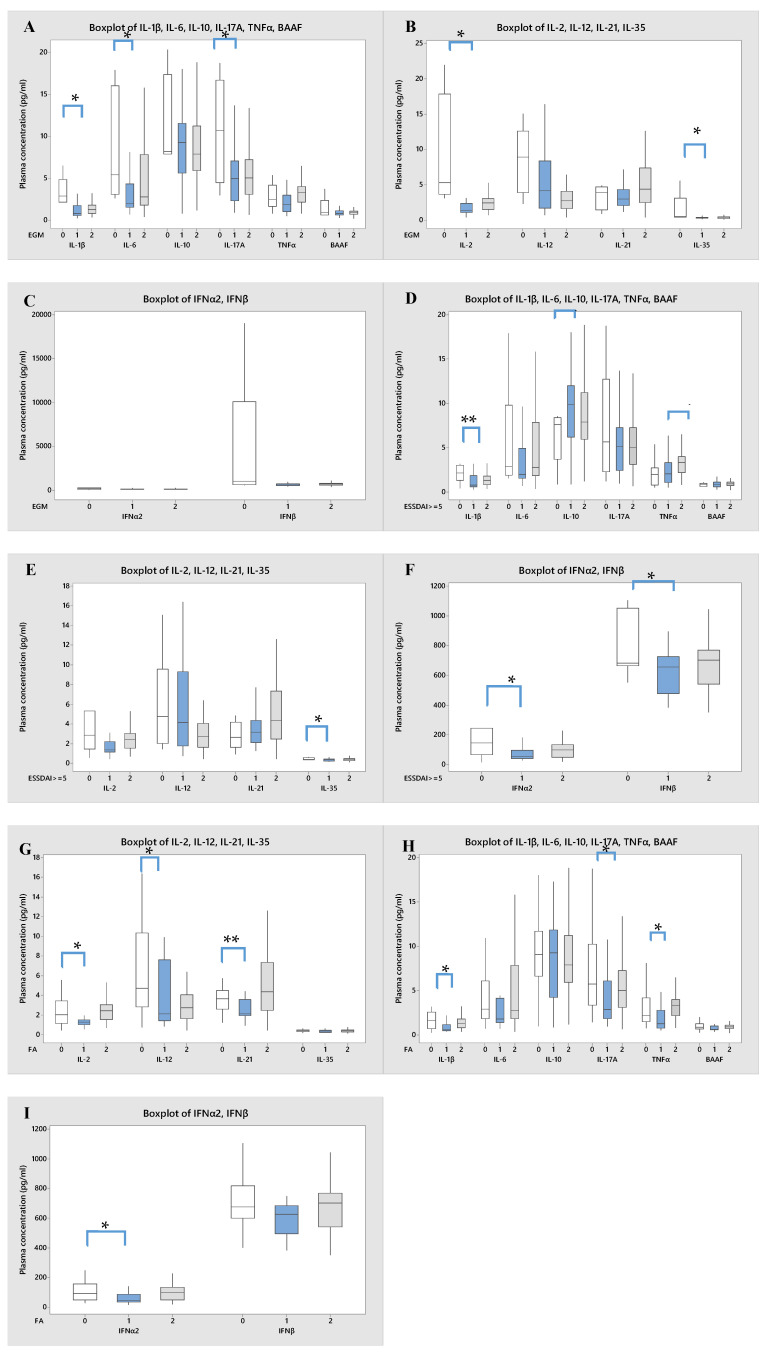
Box-plot representation showing a comparison of cytokine levels. (**A**–**C**). Correlation between cytokine levels and presence of EGMs, where 0 = patients with no EGMs, 1 = patients with EGMs, and 2 = control group. (**D**–**F**). Association between cytokine levels and ESSDAI, 0 = patients with ESSDAI <5, 1 = patients with ESSDAI ≥ 5, and 2 = control group. (**G**–**I**), Association between cytokine levels and fatigue in patients, 0 = no fatigue, 1 = confirmed fatigue, and 2 = control group. Data are presented as mean (pg/mL) ± SD. * *p* < 0.05, ** *p* < 0.001.

The association between plasma cytokine levels in patients with pSS with or without EGMs revealed significantly low IL-1β (*p* = 0.014), IL-2 (*p* = 0.035), IL-6 (*p* = 0.014), IL-17 (*p* = 0.043), and IL-35 (*p* = 0.010) levels in patients with EGMs (Figure 3A–C). Additionally, patients who had low disease activity displayed decreased IL-10 (*p* = 0.018) and high IFN-α ( *p* = 0.049), IFN-β (*p* = 0.049), IL-1β (*p* = 0.006), and IL-35 (*p* = 0.032) levels compared to patients with high disease activity. Nevertheless, there was no significant difference in the cytokine profile between controls and patients with low disease activity, except at the TNFα level (*p* = 0.013) (Figure 3D–F). With regard to ESSPRI, there was no significant association in cytokine levels between patients with an ESSPRI of <5 or ≥5. However, a negative association between a positive fatigue score and IL-1β (*p* = 0.010), IL-2 (*p* = 0.037), IFN-α (*p* = 0.025), TNFα (*p* = 0.030), IL-17 (*p* = 0.029), IL-12 (*p* = 0.046), and IL-21 (*p* = 0.005) levels was found (Figure 3G–I).

## 4. Discussion

In the present study, we investigated for the first time the association between proinflammatory and anti-inflammatory cytokine levels in Saudi patients with pSS and EGMs, ESSDAI, ESSPRI, and fatigue in an effort to assess the immune pathways involved in these different phenotypes. Moreover, we evaluated the difference between cytokine profiles in patients with low disease activity scores and controls. Our results showed similar cytokine levels in patients using ISMs and those who did not; this result might be affected by the small sample size included in this study. However, levels of both TNFα and IL-21 were lower in patients than those in in healthy controls. The main reason for this decrease was the effect of drug administration. Such treatment with ISMs may affect levels of inflammatory cytokines, especially proinflammatory cytokines, such as TNFα and IL-21. This result is in line with those of other studies that confirmed the inhibitory effect of ISMs on cytokine levels [25,26]. Nevertheless, previous studies on patients not using ISMs reported that TNFα [14,15,27] and IL-21 [28,29] levels were elevated in patients with pSS compared to those in controls. Moreover, TNFα levels are elevated in the saliva [29] and tears of pSS patients [30,31]. One study published by López-Villalobos et al. showed that a higher cytokine profile was associated with more severe disease as measured via outcome measures such as ESSDAI and such a measure is mainly driven by extra-glandular manifestations [32]. Weaver et al. proposed the hypothesis that skewing inflammatory responses from Tregs toward Th17 or T helper 1 (Th1) responses induces immune disease progression via T helper 2 (Th2) cells [33]. This can explain the decreased TNFα level, which is important for inducing apoptosis, as well as the IL-21 level, which induces B cell proliferation and differentiation [34] in this study.

In contrast, our results showed that IL-12P40 levels were increased in patients with pSS compared to those in controls, which is in line with the results of other studies [14,15] and can be attributed to the fact that IL-12 (known as IL-12p70) is a proinflammatory cytokine that promotes the differentiation of Th1 cells [35]. IL-12p70 is a heterodimeric structure composed of p35 and p40 subunits, and IL-12p40 may act as an antagonist of IL-12p70 function via the inhibition of the generation of cytotoxic T lymphocytes in vitro [36]. Another study found patients with high serum heterodimeric IL-12p70 levels to be less sensitive to B-cell-targeted therapy [37].

Overall, the most important clinical marker of disease activity evaluation in pSS is the presence of EGMs. We found that 36 (87.8%) patients had one or more EGM, which is similar to the frequency of 80% of EGMs in 41 patients with pSS reported by Zazzetti et al. [38]. Nevertheless, there was a distinct difference between our results and those of the Spanish cohort reported by Ramos-Casals et al. in 2014: among 921 patients, 40% had systemic involvement [39].

In our study, there was a negative association between EGMs and peripheral cytokine levels in patients with pSS. This reduction in EGM incidence might be explained by the findings of the study reported by Demarchi et al., who compared the rate of EGM incidence in patients with pSS receiving and not receiving hydroxychloroquine therapy. These authors found that 52% of patients had at least one EGM during the course of the disease; additionally, EGMs were less frequent in patients receiving HCQ therapy (36.5% vs. 63.5%, *p* < 0.001) [40]. 

According to our results, patients who had low disease activity had decreased cytokine levels compared to those with high disease activity. This indicates that cytokines may be considered systemic inflammatory markers reflecting pSS patient status. This is in line with the results of many recent studies that found an association between the ESSDAI score and cytokine levels [41,42,43].

Correspondingly, our study confirmed a negative association between fatigue and cytokine levels in patients with pSS. Regarding the anti-inflammatory negative feedback loop, Tripp et al. reported that, in the case of pSS, there is constant exposure to immune challenge, which results in chronic inflammation and an inappropriate anti-inflammatory response. These authors hypothesized that this excessive immune regulatory response could alter the adaptive behavioral response, resulting in persistent and pathological chronic fatigue [14].

Limitations of this study include the small sample size from a single center, the fact that age was statistically different between patients and controls, selection bias, with most EGMs being pulmonary and with no inadequate representation of other manifestations, and the limited number of cytokine assessments, which may have led to pertinent ones being overlooked. Moreover, cryoglobulin levels at the time of study inclusion were not available.

## 5. Conclusions

In conclusion, proinflammatory cytokine levels were significantly different in pSS patients compared to those in healthy controls. There was a significant association between cytokine levels and EGMs, ESSDAI, and fatigue, and patients with low disease activity had a cytokine profile similar to that of healthy controls. Despite these promising results, many questions remain unanswered. Further assessments of the ISM effect on disease phenotype in pSS patients with Arab ancestry, which has not been adequately described, are needed.

## Figures and Tables

**Table 1:** Demographics and outcome measures of the study population.

Variable	pSS Patients (*n* = 41) (mean ± St. dev.)	Control (*n* = 71) (mean ± St. dev.)
Age	58.76 ± 12.7	48.87 ± 14.49 *
ESSDAI	9.95 ± 7.73	-
ESSPRI	5.17 ± 2.4	-
PGA	5.2 ± 2.37	-
PhGA	4.46 ± 2.06	-

ESSDAI: EULAR Sjogren’s syndrome disease activity index, ESSPRI: EULAR Sjogren’s syndrome patient reported outcome, PGA: patient global assessment, PhGA: physician global assessment. * *p* < 0.001.

**Table 2:** Levels of different cytokines in patients with pSS and the control group.

Cytokines (pg/mL)	*N*	Patients with pSS	*N*	Control Group	r	*p*-Value
		Mean ± St. dev		Mean ± St. dev		
IFN-α	41	115.2 ± 132.1	71	100.42 ± 48.24	−0.69	0.492
IL-10	40	9.57 ± 6.01	71	8.97 ± 5.27	−0.53	0.595
IL-1β	41	1.6 ± 1.55	71	2.02 ± 4.04	0.79	0.432
IL-2	40	3.93 ± 7.61	71	2.94 ± 3.50	−0.77	0.444
IL-6	41	6.02 ± 11	71	10.87 ± 26.77	1.34	0.182
TNFα	41	2.54 ± 1.98	71	3.13 ± 2.15	2.16	0.034 *
IL-17	41	5.83 ± 4.29	71	5.91 ± 3.87	0.10	0.919
IL-12	41	5.88 ± 4.99	71	5.05 ± 3.13	−3.36	0.002 *
IL-21	41	3.29 ± 1.51	71	3.37 ± 1.90	4.00	<0.001 **
IL-35	41	0.50 ± 0.82	71	0.38 ± 0.13	−0.95	0.350
IFN-β	41	1215 ± 2957	71	675.91 ± 155.77	−1.17	0.250
BAFF	41	0.91 ± 0.57	71	0.94 ± 0.33	0.26	0.798

* *p* < 0.05; ** *p* < 0.001.

**Table 3:** Differences in levels of different cytokines in patients with pSS based on the status of immunosuppressant medication (ISM) intake.

Median Cytokine Levels in Patients Using ISMs		Median of Cytokine Levels in Patients not Using ISMs		Chi-Square	*p*-Value
IFNα2	51.375	IFNα2	146.96	3.45	0.063
IL-10	9.27	IL-10	8.21	0.53	0.465
IL-1β	0.72	IL-1β	1.84	3.45	0.063
IL-2	1.345	IL-2	2.55	2.13	0.144
IL-6	2.045	IL-6	2.85	1.33	0.249
TNFα	2.06	TNFα	1.5	0.07	0.796
IL-17A	5.135	IL-17A	5.64	0.20	0.655
IL-12	4.15	IL-12	4.78	0.20	0.655
IL-21	3.29	IL-21	2.38	0.93	0.335
IL-35	0.37	IL-35	0.41	1.81	0.179
IFNβ	660.44	IFNβ	670.6	0.20	0.655
BAFF	0.88	BAFF	0.68	2.78	0.095

## Data Availability

The datasets generated and/or analyzed in the current study are not publicly available because of ethical concerns but are available from the corresponding author upon reasonable request.

## List of Abbreviations

BAFF	B-cell activating factor
ESSPRI	EULAR Sjögren’s Syndrome Patient Reported Index
EGMs	Extraglandular manifestations
ISMs	Immune-suppressive medications
IRB	Institutional review board
IFN-α	Interferon alpha
IFN-β	Interferon beta
IFN-γ	Interferon gamma
IL-1β	Interleukin-1 beta
IL-10	Interleukin-10
IL-12	Interleukin-12
IL-17	Interleukin-17
IL-2	Interleukin-2
IL-21	Interleukin-21
IL-4	Interleukin-4
IL-6	Interleukin-6
PGA	Patient global assessment
PhGA	Physician global assessment
pSS	Primary Sjögren’s syndrome
STAT-4	Signal transducer and activator of transcription
Th1	T helper 1
ACR/EULAR	The American College of Rheumatology/European League Against Rheumatism
ESSDAI	The EULAR Sjögren’s syndrome disease activity index
EULAR	The European League Against Rheumatism
FSS	The fatigue severity scale
TNFα	Tumor necrosis factor alpha
